# Real-time Mental Health Impact of the COVID-19 Pandemic on College Students: Ecological Momentary Assessment Study

**DOI:** 10.2196/24815

**Published:** 2020-12-15

**Authors:** Evan M Kleiman, April L Yeager, Jeremy L Grove, John K Kellerman, Joanne S Kim

**Affiliations:** 1 Rutgers, The State University of New Jersey Piscataway, NJ United States

**Keywords:** ecological momentary assessment, college students, COVID-19, anxiety, real-time, mental health, impact, student

## Abstract

**Background:**

College students’ mental health may be disproportionally affected by the COVID-19 pandemic because of the abrupt shift off campus and subsequent loss of a social network and potential long-term impact on job prospects.

**Objective:**

We sought to assess the nature of COVID-19’s mental health impact among a sample of undergraduates who were experiencing the pandemic as it occurred in real time.

**Methods:**

In total, 140 college students completed smartphone-based ecological momentary assessments of anxiety and optimism related to COVID-19 and other generic mental health variables 6 times daily.

**Results:**

Participants completed >23,750 surveys. Overall, >75% of these surveys indicated at least some level of anxiety about COVID-19. On average, the proportion of responses each day at the highest levels of anxiety about COVID-19 was 7 times greater than the proportion of responses at the highest levels of non–COVID-19–specific anxiety. Structural change analyses indicated a significant downward trend in COVID-19 anxiety after the first week of June, but even at the lowest point, >15% of the participants in the sample still reported high levels of COVID-19 anxiety each day. Participants felt more anxious about COVID-19 on days when the number of new cases and deaths due to COVID-19 were higher. When participants felt anxious about COVID-19, they also felt sad, anxious (in general), and had a greater desire to drink and use drugs. Participants felt more optimistic about COVID-19 when they received more support from others and from their university.

**Conclusions:**

This study demonstrated the widespread mental health impact that COVID-19 has had on college students.

## Introduction

### Background

The COVID-19 pandemic has had a large impact on mental health [1-4] particularly among those on the front line combating the pandemic [5,6] and those who are staying at home due to social distancing mandates and have been displaced from routine activities and social contact (eg, young children [7] and older adults [8]). One group that has received relatively less attention but whose mental health may be disproportionally and uniquely impacted by COVID-19 in the short term and long term is college students [9-11]. In the early weeks of the pandemic hitting the United States, college students faced abrupt closures of schools and subsequent displacement from their on-campus housing. The economic downturn in the later phases of the pandemic influenced students’ ability to afford returning to campus (eg, if students’ on-campus jobs were eliminated), to obtain internships, and to procure stable employment after graduation. These impacts place a large mental health burden on college students.

Some empirical research on college student mental health during COVID-19 has been published. However, with few exceptions [12], this work has been predominantly cross-sectional [13,14]. Although it is useful to determine the impact of the pandemic at one point in time, this research does not allow us to understand the dynamics of the mental health impact of COVID-19. The COVID-19 pandemic was (and still is, as of December 2020) evolving daily and the impact on mental health seen one day could be quite different from what was seen on another day. Accordingly, the goal of this study is to explore how the rapidly changing impact of COVID-19 on mental health evolves over time using high-resolution, real-time data collection.

### Research Questions

#### What is COVID-19’s Impact on Mental Health?

We assessed two COVID-19–specific mental health variables: anxiety about COVID-19 and optimism about COVID-19. Given the rapidly changing nature of the COVID-19 pandemic, we wanted to capture, over time, the severity and reach of the mental health impact (eg, “What percent of students experience severe COVID-19 anxiety throughout the day?”). We also wanted to see how these COVID-19 variables compared to other non–COVID-19–specific mental health variables, such as anxiety or worry. This allowed us to determine whether the pandemic’s mental health impact differed (eg, in rates, severity) from other mental health experiences that college students may face. We had no specific a priori hypotheses about this question, since it is primarily descriptive.

#### What Factors Characterize Periods of Anxiety and Optimism About COVID-19?

We were interested in the role of day-level contextual factors that could characterize days when the mental health impact of COVID-19 would be particularly severe. Such information can inform when and how to prevent or reduce any deleterious effects of COVID-19 on mental health. We focused on constructs that reflected exposure to negative news about COVID-19, including the number of new COVID-19 cases and deaths due to COVID-19 and the amount of COVID-19–related media consumed each day. We expected that participants would report higher anxiety and lower optimism on days when the numbers of new COVID-19 cases and/or deaths were higher and when they consumed more news related to COVID-19. One cross-sectional study conducted during the pandemic found that greater social media usage (not specific to COVID-19) was associated with higher anxiety [15].

We were also interested in the role that interpersonal and institutional support regarding COVID-19 could have in blunting the adverse mental health effects of COVID-19. Natural disasters have a lesser impact on the mental health of those who perceive more social support [16,17]. We assumed the same may be true here: those who perceive more support may be less impacted by COVID-19. Social support may be particularly relevant to undergraduates during the pandemic given that their on-campus social networks were quickly and unexpectedly disrupted. We also expected that support from institutions, especially the university, may play a role in how students respond to the pandemic. During the pandemic, students were faced with incredible uncertainty about whether they would be able to receive the same education remotely as they would in person, as well as when and how they would be able to return to campus. Institutions like universities play a large role in providing support around this uncertainty (eg, by creating resources to help students cope) and thus we expected that greater perceived institutional support would be associated with a reduced impact on mental health. To our knowledge, this is the first study to assess daily perceptions of how supported students feel from institutions like the university they attend.

#### What Are the Proximal Consequences of Anxiety and Optimism About COVID-19?

Although anxiety and optimism about COVID-19 are relevant end points, it is likely that COVID-19’s mental health impact does not stop at anxiety and optimism specific to COVID-19. We thought it was likely that anxiety and optimism about COVID-19 would lead to other proximal mental health consequences, such as extended periods of anxiety and attempts to cope, possibly in maladaptive ways. Understanding these consequences further allows us to characterize COVID-19’s mental health impact. Specifically, we were interested in whether anxiety and optimism about COVID-19 would have any appreciable impact on other mental health variables (ie, ratings of sadness or anxiety), health behavior variables (ie, urge to drink and urge to use drugs), and interpersonal variables (ie, feeling connected to others). We hypothesized that COVID-19 anxiety would be positively associated with adverse mental health consequences (sadness, anxiety, urge to drink, urge to use drugs) and negatively associated with social connection. We expected the opposite pattern for optimism about COVID-19.

## Methods

### Recruitment

Included in this manuscript are 140 participants from an ongoing study who were recruited between April 24 and May 26, 2020. The sample of 140 came from a total sample of 143 participants who completed the consent form and baseline; of the 143 participants, 3 did not complete the smartphone monitoring portion and were thus excluded from the study.

### Procedure

#### Recruitment and Baseline

Participants were recruited remotely (stay-at-home orders were in place before the beginning of the study) from the undergraduate psychology pool and several large psychology classes. Participants first completed a screener for primary study inclusion criteria (aged ≥18 years, compatible smartphone, willing and able to do the surveys). They then completed a baseline assessment that assessed demographics and other constructs not used in this manuscript. After baseline, they received instructions for the app (MetricWire) we used to send the smartphone surveys.

#### Smartphone Surveys

Using their smartphones, participants completed a brief (<5 minutes) ecological momentary assessment (EMA) assessing general affect at the present moment (eg, anxious, worried) on a scale from 0 (not at all present) to 10 (very much), 6 times per day at random times within prespecified windows. This survey also included a COVID-19–specific anxiety question ("How worried or anxious are you about the coronavirus outbreak?") that was answered on a scale from 0 (not at all) to 5 (very much).

In addition to the surveys assessing the current moment, the last assessment of each night included questions that asked participants to reflect over the entire day. In this study, we used 3 COVID-19–specific items that asked participants to rate their answers to the following questions on a scale from 0 (not at all) to 5 (very much).

1. How frequently did you see or read news or media about coronavirus today?
2. Which of the following best describes how supported you feel by friends, family, or other individuals you know in dealing with the coronavirus outbreak?
3. Which of the following best describes how supported you feel by groups, organizations, or institutions you belong to in dealing with the coronavirus outbreak (eg, school, workplace, religious institution, community organization)?

This assessment also measured optimism about COVID-19 (“What best describes how optimistic you feel about the coronavirus outbreak?”) on a scale from 1 (very pessimistic) to 5 (very optimistic).

#### Compensation

For the baseline, participants were given the option to receive a US $15 Amazon gift card or extra class credit. For the EMA portion, participants were paid $0.25 (in the form an Amazon gift card) for each of the surveys except for the longer nightly survey, for which they were paid $0.50. If participants completed ≥4 surveys each day, they received a $0.50 bonus. In total, participants had the opportunity to be paid as much as $141.

#### COVID-19 Data Extraction

We used data on the number of new COVID-19 cases and deaths from COVID-19 for each day in the United States and New Jersey (nearly all participants resided in New Jersey during the study). These data were obtained using the *COVID-19* R package [18], which uses the COVID-19 Data Hub to obtain gold-standard COVID-19 data from the repository hosted by the Center for Systems Science and Engineering at Johns Hopkins University [19].

### Statistical Analysis

#### What is COVID-19’s Impact on Mental Health?

As COVID-19 anxiety was measured at a higher resolution than COVID-19 optimism (momentary versus daily), we primarily focused on anxiety for the descriptive analyses. To explore trends over time, we calculated for each day the percentage of responses at the highest level of the scale (≥4 out of 5 for COVID-19 anxiety, ≥8 out of 10 for all other momentary variables). We conducted a structural change model using the *strucchange* package [20,21] to derive breakpoints where COVID-19 anxiety increased or decreased. To describe the mental health impact of COVID-19 across the sample, we calculated descriptive statistics including the frequency of non-zero and most-severe responses and the intraclass correlation (ICC) showing the amount of variability from observation-to-observation (versus person-to-person). To put the COVID-19–specific variables into context, we calculated similar statistics for two similar EMA questions: ratings of general worry and anxiety.

#### What Factors Characterize Periods of Anxiety and Optimism About COVID-19?

We were interested in which day-level contextual variables affected COVID-19 anxiety and optimism, specifically the following: the number of new cases/deaths announced each day in New Jersey (nearly all participants were in the state), perceived support related to COVID-19 from others and from organizations, and time spent consuming news media related to COVID-19. We conducted separate models with daily average level of COVID-19 anxiety (ie, the mean of all ratings for each participant on each day) and daily level of COVID-19 optimism as outcome variables.

All analyses for this aim have two levels: days (level-1) nested within people (level-2), which were tested using the *lme4* [22] R package. We centered on person means of all self-reported predictors (support from others and organizations, frequency of upsetting news about COVID-19).

#### What Are the Proximal Consequences of Anxiety and Optimism About COVID-19?

We conducted contemporaneous and temporal multilevel models for each set of associations between COVID-19 anxiety or optimism and one of the outcome variables (anxious, sad, desire to use drugs, desire to use alcohol, feeling close/connected to others). We restricted temporal analyses regarding COVID-19 anxiety to response pairs that were spaced by <6 hours. Since COVID-19 optimism was assessed once daily, we aggregated the momentary variables to create a daily average for each construct. We restricted the temporal analyses to pairs of consecutive days. As in the prior aim, we used the *lme4* package with person-centered predictors.

## Results

### Overview

Between April 24 and July 19, 2020, 140 participants contributed 23,793 data points. Participants answered at least one survey on 5728 days (ie, each participant, on average, gave at least one survey response on a total of 40.91 days). This led to a response rate of 69.23% of surveys completed across those days.

### Demographics

Among the 140 participants, 77.6% (n=109) were female and the average age was 19.98 years (SD 1.61, range 18.44-33.23). Regarding race, 48.59% (n=68) of the sample identified as White, 36.62% (n=51) Asian, 7.04% Black/African American, 1.41% American Indian/Alaskan Native, and the remaining 6.34% identified as multiple or other races. At the baseline assessment, 69.2% of the sample lived either on campus in the dorms or immediately off-campus and 30.1% of the sample were commuter students (eg, living with family). Although none of the participants reported COVID-19 diagnoses at baseline, 1.4% of the sample reported experiencing symptoms of COVID-19. A large proportion of the sample reported knowing someone who had a COVID-19 diagnosis or symptoms; overall, 12.1% of the sample knew someone who had been diagnosed with COVID-19 and 5.7% of the sample knew someone who was experiencing potential COVID-19 symptoms.

### What is COVID-19’s Impact on Mental Health?

**Figure 1:**
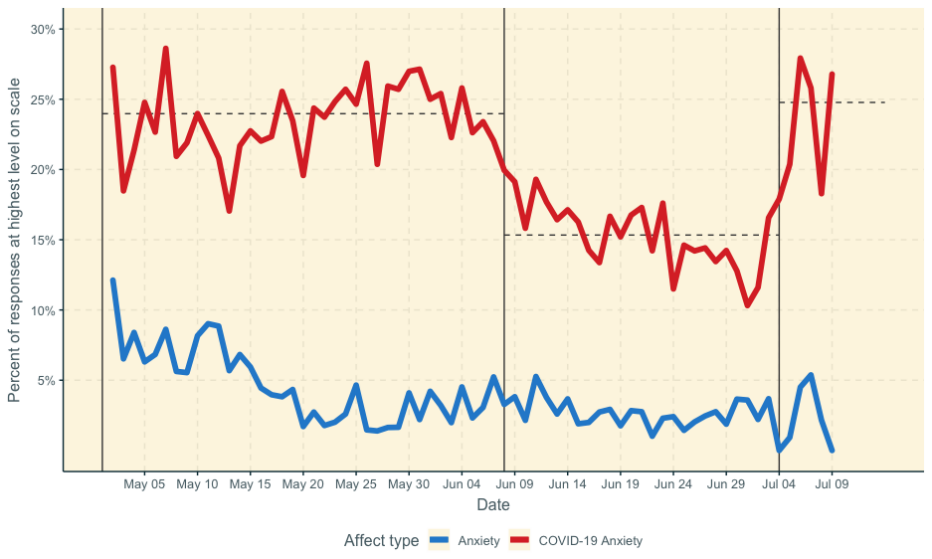
Daily proportion of responses at highest levels of anxiety about COVID-19 and non–COVID-19–specific anxiety. Vertical lines indicate breakpoints in COVID-19 anxiety from the structural change model. Horizontal lines indicate mean during each segment.

Figure 1 shows the percent of daily responses each day that were at the highest levels of the scale for anxiety about COVID-19 and non–COVID-19–specific anxiety. The proportion of the sample endorsing the highest levels of COVID-19 anxiety on any given day was, on average, 6.89 (SD 4.12) times the rate of those endorsing the highest levels of anxiety.

The structural change model identified three segments with breakpoints at the 38th (June 8, 2020) and 64th (July 4, 2020) day. The first segment, from May 3 to June 8, 2020, was statistically flat (*b*<0.001, *t*=–1.16, *P*=.26, *M*=23.5% of responses were at the highest level of the scale). The second segment, from June 9 to July 3, 2020, showed a decrease in worry (*b*=–0.002, *t*=–3.69, *P*=.001, *M*=15.3%). The third segment, from July 4 to 9, 2020, was also statistically flat (*b*=0.009, *t*=–0.53, *P*=.62). However, the average proportion of people who had high levels of anxiety about COVID-19 each day during this third segment was significantly higher than the prior period (24.78% versus 15.3%, *t*=3.78, *P*=.006).

Multimedia Appendix 1 shows participant-level data across the study. These data followed the same trend as the day-level data. Across the study, significantly more responses were non-zero (*χ*^2^_1_=5023.4, *P*<.001) for COVID-19 anxiety (78.5% of all responses were >0) than for “anxious” (47.0%). Similarly, a greater number of people reported the highest levels of COVID-19 anxiety (≥4 out of 5) at least once in the study (78.5%) than they did the highest levels of “anxious” (51.7% of participants reported a score of ≥8 out of 10 at least once). Nearly 75% of the variability in ratings of COVID-19 anxiety occurred from response-to-response (ICC=0.74, 95% CI 0.70-0.79). This ICC is significantly higher (ie, because the confidence intervals do not overlap) than the within-person variability for ratings of “anxious” (ICC=0.51, 95% CI 0.45-0.57).

### What Factors Coincide With Anxiety and Optimism About COVID-19?

The left column of Table 1 shows which factors coincided with daily anxiety about COVID-19. Anxiety about COVID-19 was positively associated with the number of new cases announced in the state each day and the amount of news consumed relating to COVID-19. Counterintuitively, perceived support from others regarding COVID-19 was positively associated with daily anxiety about COVID-19. The right column of Table 1 shows which factors coincided with daily COVID-19 optimism. Daily optimism about COVID-19 was positively associated with both support from others and organizations, but unassociated with all other variables.

**Table 1:** Result of multilevel models showing which factors coincide with anxiety and optimism about COVID-19.

Factors		Daily anxiety about COVID-19			Daily optimism about COVID-19	
		Value	*P* value	Value		*P* value
**Predictors**						
	Intercept, β (95% CI)	0.079 (–0.076 to 0.234)	.32	0.015 (–0.099 to 0.128)		.80
	New cases in New Jersey, β (95% CI)	0.021 (0.008 to 0.034)	.002	–0.004 (–0.024 to 0.017)		.73
	New deaths in New Jersey, β (95% CI)	–0.005 (–0.017 to 0.006)	.37	–0.015 (–0.033 to 0.004)		.12
	Support regarding COVID-19 from others, β (95% CI)	0.027 (0.014 to 0.040)	<.001	0.348 (0.329 to 0.368)		<.001
	Support regarding COVID-19 from organization, β (95% CI)	0.008 (–0.005 to 0.021)	.21	0.092 (0.073 to 0.112)		<.001
	Frequency of upsetting news regarding COVID-19, β (95% CI)	0.085 (0.073 to 0.097)	<.001	–0.010 (–0.029 to 0.009)		.30
**Random effects**						
	σ^2^	0.39	N/A^a^	0.68		N/A
	τ_00_	2.04 _Participant_	N/A	0.74 _Participant_		N/A
	Intraclass correlation	0.84	N/A	0.52		N/A
	Marginal *R*^2^ / conditional *R*^2^	0.010 / 0.841	N/A	0.152 / 0.593		N/A

^a^N/A: not applicable.

### What Are the Proximal Consequences of Anxiety and Optimism About COVID-19?

#### Consequences of Anxiety About COVID-19

The first row of Table 2 shows the positive contemporaneous associations between anxiety about COVID-19 and ratings of anxiety, sadness, urge to drink, and urge to use drugs. The second row of Table 2 shows the temporal associations between anxiety about COVID-19 (at time T) and ratings of anxiety, sadness, urge to drink, and urge to use drugs (at time T+1). There was no association between anxiety about COVID-19 and desire to use drugs.

#### Consequences of Optimism About COVID-19

The third row of Table 2 shows the negative contemporaneous associations between optimism about COVID-19 and ratings of sad and urge to drink and the positive contemporaneous association between optimism about COVID-19 and feeling close/connected. There were no associations between optimism about COVID-19 and feelings of anxiety and the urge to use drugs. The fourth row of Table 2 shows the temporal associations between optimism about COVID-19 (at time T) and our outcome variables of interest (at time T+1). We found no significant temporal associations.

**Table 2:** Contemporaneous and short-term temporal associations between anxiety/optimism about COVID-19 and mental health, behavioral health, and connectedness dependent variables^a^.

Predictor	Dependent variable: anxiety		Dependent variable: sadness		Dependent variable: desire to use alcohol		Dependent variable: desire to use drugs		Dependent variable: close/connected	
	β (95% CI)	*P* value	β (95% CI)	*P* value	β (95% CI)	*P* value	β (95% CI)	*P* value	β (95% CI)	*P* value
COVID-19 anxiety (contemporaneous)	0.055 (0.044 to 0.066)	<.001	0.054 (0.043 to 0.064)	<.001	0.037 (0.026 to 0.049)	<.001	0.019 (0.009 to 0.029)	<.001	0.015 (0.005 to 0.025)	.003
COVID-19 anxiety (temporal)	0.033 (0.021 to 0.046)	<.001	0.035 (0.023 to 0.047)	<.001	0.023 (0.010 to 0.036)	.001	0.011 (–0.001 to 0.022)	.07	0.015 (0.003 to 0.027)	.02
COVID-19 optimism (contemporaneous)	–0.004 (–0.024 to 0.017)	.72	–0.027 (–0.046 to –0.007)	.007	–0.024 (–0.046 to –0.002)	.03	0.007 (–0.009 to 0.023)	.40	0.015 (0.003 to 0.032)	.01
COVID-19 optimism (temporal)	–0.001 (–0.023 to 0.021)	.93	–0.003 (–0.023 to 0.017)	.78	0.00 (–0.023 to 0.022)	.97	0.013 (–0.004 to 0.031)	.13	0.007 (–0.012 to 0.026)	.47

^a^Each row in each section (anxiety/optimism and contemporaneous/temporal) represents separate analyses. As optimism was assessed on the daily level, we aggregated (averaged) the momentary dependent variables for each day.

## Discussion

### Overview

We sought to better understand the nature of the mental health impact of the COVID-19 pandemic on a sample of college students as they were experiencing the pandemic in real time. We had three key goals: (1) to describe the variability of mental health variables related to COVID-19, (2) to identify which day-level factors coincided with anxiety and optimism about COVID-19, and (3) to identify the downstream consequences of the mental health impact of COVID-19. We discuss below the specific findings related to each of our three key questions and then conclude with a more general discussion of the study.

### What is COVID-19’s Impact on Mental Health?

The findings regarding the frequency of anxiety about COVID-19 demonstrated that, compared to other similar states like nonspecific anxiety and worry, anxiety about COVID-19 in particular happened more often (non-zero responses occurred more than 75% of the time compared to less than 45% of the time for the other affect states), for more people (almost 80% of people reported the highest levels of COVID-19 anxiety at some point versus almost 50% of people reporting the highest levels of nonspecific anxiety), and changed more frequently throughout the day. This is notable because it means that students who otherwise may not be experiencing much distress are now experiencing at least some level of distress related to COVID-19. Although we did find a decrease in overall anxiety as time went on, even at its lowest point in the middle of June, more than 15% of all responses each day indicated severe anxiety about COVID-19. This increased anxiety among students could lead to a greater demand for counseling services as students adapt to the new reality of campus life during COVID-19. Methodologically, these findings are important because studies that assess only “anxiety” but not anxiety specifically related to COVID-19 may risk missing a sign of impaired functioning among students during this time.

### What Factors Coincide With Anxiety and Optimism About COVID-19?

Participants reported greater anxiety about COVID-19 on days when the number of new cases announced in the state were higher and when they consumed more upsetting news specific to COVID-19. Some factors (the number of cases, deaths) are not under students’ control, while others are (eg, the frequency of watching upsetting news about COVID). This could point to the need to consume news in smaller quantities to avoid it having an undue impact on mental health [23].

Our findings highlighted the role of support during the pandemic, especially from organizations (eg, the university), which was positively associated with optimism about COVID-19. It should be noted, however, that literature on traumatic events [24] cautions that when universities do respond to events like this, care should be taken to not assume that all students are experiencing the event in the same way or experience it adversely at all. Moreover, although feeling more optimistic about COVID-19 may have potential mental health benefits, such optimism, especially if unrealistic, may carry potential health risks. There is a long history of literature on the idea that unrealistic optimism leads people to underestimate their risk of a negative health outcome and thus to engage in risky health behaviors [25,26]. Thus, in this case, those who are more optimistic about COVID-19 may underestimate their risk of exposure, leading them to engage in behaviors that would actually increase their risk of exposure.

Interestingly, we found that students feel more (rather than less) anxious on days when they perceived more support about COVID-19 from others. This could indicate that days of high anxiety are times when others would need to be more responsive. In other words, it may be that anxiety about COVID-19 leads to more support from others (to address that anxiety) rather than the other way around.

### What Are the Proximal Consequences of Anxiety and Optimism About COVID-19?

We found that feeling anxious about COVID-19 now is associated with distress (anxiety, sadness) and negative health behaviors (the desire to drink and use drugs) both in the moment and a few hours later. The increased urge to drink and use drugs may indicate an elevated likelihood of coping with this distress in maladaptive ways. We also found that optimism about COVID-19 was similarly associated with most same-day consequences but was not associated with any of the outcome variables when examined one day later. The lack of next-day effects for COVID-19 optimism could reflect some difference between optimism and anxiety, but it is probably more likely that this reflects a difference in the timescale of interest (ie, we measured anxiety related to COVID-19 six times daily and optimism once daily, about the entire day). This could mean that the direct mental health impact of COVID-19 is most relevant in the short term. This echoes the earlier findings in this study that these constructs are highly variable over a short period of time. It should be noted that although the direct impact is only present for a few hours, the cumulative effect of anxiety associated with COVID-19 could be deleterious in the long term in ways not assessed here (eg, through increased drinking over months, a transition into more general anxiety).

### Limitations and Strengths

There were several limitations to this study that should be acknowledged. First, because this study began during the COVID-19 pandemic, we were not able to establish a “baseline” for the level of worry or anxiety that students typically experience. Second, although we had >23,750 responses, these responses only came from 140 students at one university. Although the sample was ethnically and racially diverse, the experiences of these students may not generalize to all students. There were several strengths of this study. It is the most fine-grained observation of COVID-19’s mental health impact to date and one of the few studies to explicitly collect data on COVID-19–relevant constructs (eg, anxiety specifically about COVID-19).

### Conclusions

There are several tangible recommendations that come from this work. Students are anxious about this pandemic and the university may be a key source of support during this time. College counseling centers may need to provide COVID-19–specific coping skills, which would be particularly useful for those students who are not typically anxious (and for whom coping skills for general anxiety/worry might not resonate). Moreover, it is possible that substance use behavior could increase in the context of a crisis and there is a need to assess for this as the pandemic continues. Finally, even after the pandemic is under control, there are long-lasting consequences that may continue to contribute to anxiety among college students (eg, finding a job after college, paying for college after the economic impact of COVID-19); further research is needed on these consequences. Such research will inform universities’ long-term recovery plans, which may need to help students address this anxiety.

## Appendix

Multimedia Appendix 1 COVID-19 anxiety and non–COVID-19–specific anxiety by participant. Only participants with >50 responses are shown. Anxiety about COVID-19 was multiplied by 2 because it was on a 0-5 scale and not a 0-10 scale. Multilevel correlation between COVID-19 anxiety and non–COVID-19–specific anxiety was small (*r*=.11, *P*<.001).

## Abbreviations

EMA: ecological momentary assessments
ICC: intraclass correlation
