# Supplementary figures and images for: Real-time Mental Health Impact of the COVID-19 Pandemic on College Students: Ecological Momentary Assessment Study
Source: JMIR Ment Health. 2020 Dec 15;7(12):e24815. doi: 10.2196/24815 (PMC7744138; doi:10.2196/24815)

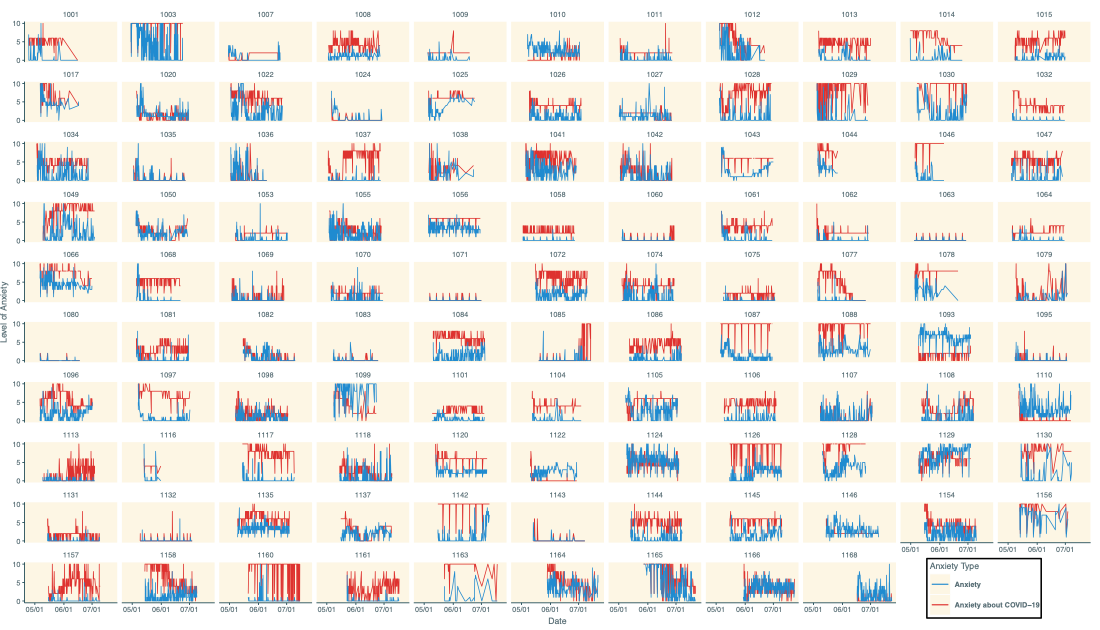

Supplement: Multimedia Appendix 1 [file mental_v7i12e24815_app1.png]
